# “The Dop System of Alcohol Distribution is Dead, but It’s Legacy Lives On….”

**DOI:** 10.3390/ijerph16193701

**Published:** 2019-10-01

**Authors:** Philip A. May, Anna-Susan Marais, Marlene De Vries, Julie M. Hasken, Julie M. Stegall, Dixie M. Hedrick, Cudore L. Snell, Soraya Seedat, Charles D.H. Parry

**Affiliations:** 1 Nutrition Research Institute, Gillings School of Global Public Health, University of North Carolina at Chapel Hill, Kannapolis, NC 27599, USA; julie_hasken@unc.edu (J.M.H.); julie_stegall@unc.edu (J.M.S.); dixie_hedrick@unc.edu (D.M.H.); 2 Stellenbosch University Faculty of Medicine and Health Sciences, Tygerberg, Cape Town 7505, South Africa; asmarais@sun.ac.za (A.-S.M.); mmdevries@sun.ac.za (M.D.V.); sseedat@sun.ac.za (S.S.);; 3 School of Social Work, Howard University, Washington 20059, DC, USA; csnell@Howard.edu; 4 Alcohol, Tobacco and Other Drug Research Unit, South African Medical Research Council, Cape Town 7505, South Africa

**Keywords:** alcohol use and abuse, epidemiology, fetal alcohol spectrum disorders (FASD), farm workers, South Africa

## Abstract

*Objective:* Determine the prevalence of Dop, a system of labor payment via alcoholic beverages, in a South African province, and its influence on maternal drinking and fetal alcohol spectrum disorders (FASD). *Methods:* Data from studies of FASD epidemiology were analyzed. *Results:* Forty-two percent to 67% of mothers reported drinking. In 1999, 5% of women reported Dop allocations in their lifetime: 14% of mothers of FASD children and 1% of controls. In 2010, 1.1% of mothers reported lifetime Dop: 1.6% of FASD mothers and 0.7% of controls. Commercial alcohol sales have replaced the Dop system. Total FASD rates remained high in rural areas in 2010 and rose in urban settings. Urban rates of total FASD surpassed rural area rates in 2010. Correlation analysis did not reveal a strong or significant, direct relationship between Dop experience and heavy drinking (*r* = 0.123, *p* < 0.001, *r*^2^ = 0.015), or the diagnosis of FASD in children (*OR* = 0.003, *p* = 0.183). *Conclusion:* Dop, as a systematic practice, is dead and does not have a direct influence on alcohol availability, heavy maternal drinking, or the probability of an FASD diagnosis. Nevertheless, today’s problematic drinking patterns were heavily influenced (shaped) by Dop and have negatively impacted the prevalence and severity of FASD.

## 1. Introduction

In the Western Cape Province (WCP) of the Republic of South Africa (SA), many people are involved in agriculture, especially viticulture, the growing of grapes, and the production of wine. SA is known globally for producing, selling, and exporting popular and fine wines of many varieties. Many workers also live or have lived on the farms. Over a span of at least two centuries, and under the system of apartheid, many farm and vineyard workers in the WCP were provided with wine as partial payment for their labor [1,2,3]. This practice was referred to as the “Dop” system, and this name euphemistically refers to having a drink or “tot” of the beverage [1]. Many public health advocates maintained that Dop still existed and was prevalent in the 1990’s, even after the advent of democracy in 1994. It was believed to present both a major public health problem, and a system of social control [2,3,4]. Furthermore, many people within SA and elsewhere believe that Dop exists today, and that the institutionalized practice of Dop allocations on some farms directly elevates problem drinking and rates of fetal alcohol spectrum disorders (FASD) to this day [5]. History holds that many workers consumed wine on a daily basis, and also engaged in heavy drinking on holidays and weekends [2,3,6]. This type of heavy weekend drinking has been found in studies conducted in other parts of South Africa [7], especially in urban areas [8]. While the Dop system has been prohibited or outlawed by a number of provincial and national statutes for many years, and general public sentiment in South Africa is overwhelmingly not supportive of Dop, many public health advocates maintained that Dop distribution remained on certain farms in the 1990’s in the WCP [6,9]. With this history in mind, we examined our epidemiological data from 1997 through 2011 to determine whether the Dop system still exists, whether it has changed in recent years, whether it has a direct impact on heavy drinking [2,4], and whether it presents a public health problem today. We were especially focused on any effect that DOP may have had on alcohol abuse among women of a childbearing age, and therefore on the prevalence of FASD. 

The five communities studied to date in SA have the highest documented general-population rates of FASD in the world [10]. From both clinical and epidemiological perspectives, SA has proven to contain communities that are vitally important for understanding the prevalence, characteristics, and etiology of fetal alcohol syndrome (FAS) and the entire FASD continuum. In one particular municipality, “Study Community 1” (SC1), and its surrounding rural areas in the WCP, five epidemiological studies of FASD prevalence and characteristics have been published [11,12,13,14,15]. The repeated sampling of this one community generated not only accurate period prevalence rates for this locale, but also the ability to monitor change over time. SC1 has also been the focus of much attention in the media and as a site for prevention and intervention activity over the past two decades. In the most recently published sample of SC1, FAS affected 59–79 children per 1000 (5.9%–7.9%), and total FASD rates were 170–233 per 1000, or 17%–23% [14]. Study Community 2 (SC2) has been researched twice in independent, school samples. Two manuscripts have been published on the prevalence and characteristics of the full continuum of FASD there [15,16]. In the most recent study of SC2, the prevalence of FAS was 89–129 per 1000 children (8.9%–12.9%) and the total FASD was 196–276 per 1000, or 20%–28%. 

Community studies on the epidemiology of FASD that were carried out by other researchers in other communities of SA [17,18,19,20] have found similarly high rates of FAS and partial fetal alcohol syndrome (PFAS), although these studies generally did not diagnose or report on the diagnosis of alcohol-related neurodevelopmental disorder (ARND). It is noteworthy that the prevalence of FASD is highest in rural areas surrounding small towns in most SA studies. Previous study findings have indicated that norms of regular binge drinking, which may have been influenced by the Dop system; a low socioeconomic status (SES); insufficient nutrition; a high fertility; and challenging conditions for child development, combine to elevate the prevalence and severity of FASD in many SA communities [21,22,23,24,25,26].

The populations studied here have changed substantially since the early 1990’s. In fact, some farms in the WCP have been transformed greatly and have instituted improved working conditions, worker compensation, and overall benefits [5]. Our research objectives in this manuscript are to determine how common Dop was from 1999 to 2011; examine temporal patterns of change; and assess the extent of Dop’s effect, direct or indirect, on public health. We specifically concentrate on drinking among women of a childbearing age and the prevalence of FASD over a recent 15-year period.

## 2. Materials and Methods 

Data for this exploration originated from seven different population-based samples, collected between 1997 and 2011, to define the prevalence and detailed characteristics of FASD in a general population sample in the WCP. These studies were active case ascertainment (ACA) studies of FASD clinical cases found among children in community public schools. Interviews were also conducted, in person, with the mothers of the study children [11,12,13,14,15,21,22,23,26,27,28]. The studies were carried out in two regional community settings of the WCP. Community A is a town of 35,000 people and surrounded by rural areas, with 15,000 people located about a one-hour drive by automobile from Cape Town. Community B is a large, separate region, located across a mountain range from Community A and located two to three hours of driving from Cape Town. Contained within Community B, there are four smaller towns and very large surrounding rural areas of vineyards and farms, with a total population approaching 65,000 people.

A three-tier system was used to screen and provide diagnoses for all children in first-grade classes in community schools who had consented to participate in the study through their parents. In Tier I, all consenting children were screened for height, weight, and head circumference, and a large random sample was chosen from all enrolled first-grade children. Every consenting child that was ≤25 centile on head circumference and/or whose height and weight participated in Tiers I and II, along with all randomly-selected children who had been selected from all enrolled children from the same classes and schools, regardless of Tier I pre-screening and size, were included. In Tier II, complete growth, development, and dysmorphology examinations were provided to the above children, both those that were small and randomly-selected [29,30]. Then, for Tier III, all randomly-selected children and those small children who were found to have physical traits characteristic of prenatal alcohol exposure and possible FASD underwent a battery of neurobehavioral tests assessing intelligence, cognitive functioning, behavior, and life skills. 

Additionally, in Tier III, the mothers of all study children were informed of our interest in identifying maternal risk factors for all FASD births, and those who consented to the maternal interview were interviewed about a variety of maternal risk factors. There were many detailed questions about alcohol consumption during the index pregnancy and in their lifetime, and the questions about alcohol access from a variety of sources, including their experience with the Dop system, were embedded in the maternal questionnaire. The questionnaire utilized a time-line-follow-back sequence, along with questions concerning diet, childbearing history and experience, residence, general health, and socioeconomic status. 

Diagnoses of the children were made according to the first revision of the U.S. Institute of Medicine (IOM) Guidelines [29]. The IOM diagnostic system was used among first-grade students in all of the samples in which the data were generated. Classification of children was based on a full consideration of the following: (1) physical growth and dysmorphology; (2) cognitive/behavioral assessments; and (3) maternal alcohol consumption. Furthermore, other known genetic and teratogenic anomalies were ruled out before a FASD diagnosis was made. Final diagnoses were made for each child in a formal, data-driven, case conference per updated guidelines and operational criteria, as suggested by the IOM committee [29].

The entire IOM continuum of FASD diagnoses is represented by four diagnoses: fetal alcohol syndrome (FAS), partial fetal alcohol syndrome (PFAS), alcohol-related neurodevelopmental deficits (ARND), and alcohol-related birth defects (ARBD) [29,31]. For FAS, a child must have a characteristic pattern of minor facial anomalies; evidence of prenatal and/or postnatal growth retardation; evidence of deficient brain growth; and if possible, confirmation of maternal alcohol consumption. For PFAS, a child must have evidence of a characteristic pattern of facial anomalies and one or more other characteristics (small head circumference and/or evidence of a complex pattern of behavioral or cognitive abnormalities, and direct or collateral confirmation of maternal alcohol consumption). For ARND, a child must have documentation of significant prenatal alcohol exposure; display neurological or structural brain abnormalities; or manifest evidence of a complex and characteristic pattern of behavioral or cognitive abnormalities not explained by genetic predisposition, family background, or environment alone. For ARBD, a child must have confirmed prenatal alcohol exposure and evidence of the characteristic pattern of facial anomalies, as well as either major malformations or a pattern or minor malformations, but a generally normal neurobehavioral performance [29]. The occurrence of ARBD is quite rare. For most of the analyses in this manuscript, all four of the specific diagnoses in the continuum of FASD are grouped together and treated as one category, denoted the total FASD.

Data analysis was performed using SPSS, Version 26 [32], for all analyses in this paper: for descriptive data and statistical tests presented in the tables and figures, for the calculation of partial correlations, and for sequential regression analysis.

## 3. Results

### 3.1. General Patterns of Alcohol, Drug, and Tobacco Use

In Table 1, alcohol, tobacco, and other drug use are profiled. In Community A, 42%–58% of mothers interviewed consumed alcohol in the 12 months prior to the interview, and in Community B, the percentage of users was higher, at 54%–67%. With only one exception in Sample 3 of Community A, 30%–39% of the mothers reported drinking in the past week. None of the average number (mean or mode) of drinking days per week in either community exceeded two, as the common pattern was to drink exclusively on weekends, usually on Friday and Saturday. The mean drinking pattern in Community A was 1.9 drinking days per week. In Community B, the mean was 1.8 drinking days per week. The average number of drinks per drinking day (DDD) was higher in Community B (5.4–5.7) than in Community A (3.9–5.5), and the overall percentage of mothers reporting drinking in Community A declined through Sample 3 (2002), but rebounded in Samples 4 and 5. Ninety-eight% (98%) to 93% of all alcohol was consumed on weekends at both sites, and the number of drinking days per week averaged 1.8–1.9 at both sites. Beer was the beverage of choice (54%–83%) in both communities, and wine was the second favorite alcoholic beverage (30%–49%). Drinking during pregnancy declined in the earlier years in Community A, but it resumed an upward trend in 2002. In Community B, an upward trend was evident from 2009 to 2010. In Community A, 41%–74% of all respondents of a childbearing age reported consuming alcohol during pregnancy, with a figure of 58%–68% in Community B. A high percentage of mothers in both communities reported smoking cigarettes in the past 30 days (77%–83%), but the number of cigarettes per day was quite low in terms of worldwide smoking averages (4.7 to 9.8). Drug use was very low in both communities, with the exception of 2.8% in Community B in 2011, but drug use was well below 2% in all other samples in both communities. Drug use appeared to increase in recent data among mothers in both communities. 

### 3.2. Dop Prevalence over Time by Sample and Residence

**Figure 1:**
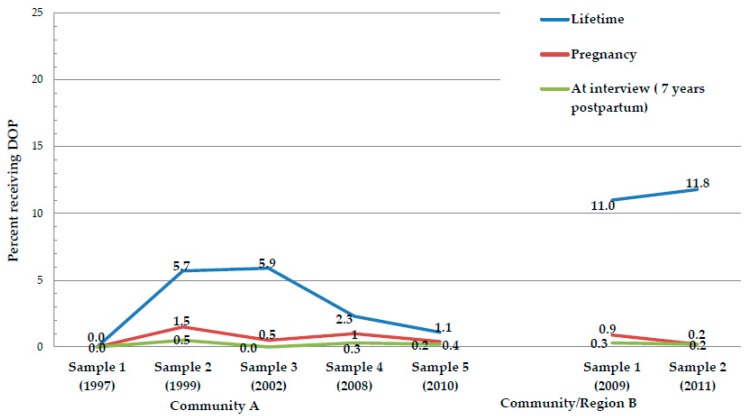
Percent receiving Dop in their lifetime, during pregnancy, and 7-years postpartum in the study community.

Table 2 and Figure 1 indicate that receiving alcohol via the Dop system in one’s lifetime was less than 6% in Community A and 11.0%–11.8% in Community B, which was the more rural region. Less than 1.5% reported receiving Dop during pregnancy, and in most samples, less than 1% reported Dop allocations. The practice of Dop decreased from the first samples to the most recent and the decline in lifetime use was statistically significant in Community A. In every sample, 0.5% or less reported receiving Dop around the time of their interview. 

**Figure 2:**
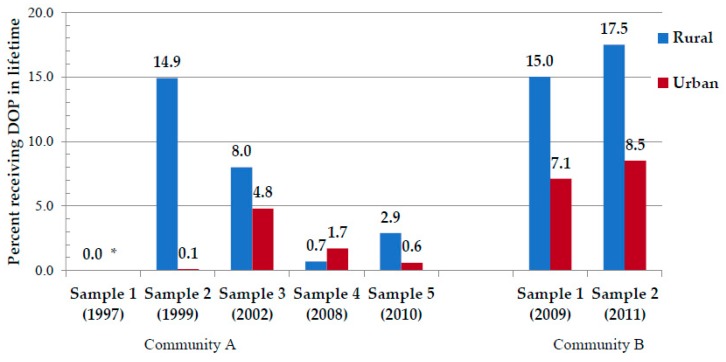
Percent receiving Dop in their lifetime by rural and urban residence. * Lifetime question not asked in Sample 1.

In Figure 2, a comparison of rural vs. urban residence is presented. Drinking was historically more common in the rural areas and on farms during the Dop system era. This pattern of heavy drinking in the rural areas continued for the entire 15 years covered by the data. In earlier samples of Community A, more rural residents reported receiving Dop allocations, although the practice of Dop declined from 14.9% of rural mothers in Sample 2 who received Dop in their lifetime to 2.9% of rural mothers in Sample 5. In Community B, rural residents also reported more lifetime experience with Dop, but given the more rural, highly agricultural, and remote nature of this region, lifetime experience with Dop was higher (15.0%–17.5%) in samples in 2009 and 2011. However, current Dop percentages were reported to be low in Community A (<0.05%) (as seen in Figure 1).

Table 3 presents the percentage of Dop received by mothers of children with different diagnoses. In each sample, lifetime Dop use was higher for mothers of children with FASD in Community A, and this was statistically significant for samples 2 and 4. In samples 1 and 2 in Community B, the mothers of Children with FASD reported significantly more lifetime experience with Dop. There was only one significant difference between mothers of children with FASD and controls for a recent time period during pregnancy, in Sample 1 (2009) for Community B. At the time of interview, no significant differences were found. 

Table 4 data indicate that women forty years of age and older had the highest overall lifetime experience with Dop (*p* < 0.001) and that Dop use declined significantly in recent years. There was no significant difference by age category in Dop use percentage during pregnancy, at interview (current use), and by rural residence. Taken together, these data indicate that Dop was rarely reported to have been received during pregnancy at any time over the 15-year period, it was rarely received in recent years, and Dop was still most common in rural areas over the 15-year period. 

Different drinking patterns were evident in rural vs. urban areas, as presented in Table 5. In rural areas, a significantly higher percentage of women reported using alcohol in their lifetime, the past 12 months, the past 30 days, and the past week. Calculated for only those mothers who reported drinking in the past year, the total number of drinking days per week, drinks per drinking day (DDD), drinks consumed on weekends, and drinking during pregnancy were all significantly higher in the rural areas. Rural mothers reported drinking 12.3 DDD vs. 8.4 DDD for urban mothers. Additionally, 70.6% of rural mothers and 49.2% of urban mothers reported drinking during pregnancy. The number of drinking days per week was not significantly different. Both rural and urban women who drank, did so in a similar pattern, for 1.9 to 1.8 days per week and mostly on weekends. Therefore, higher levels of drinking prevalence and quantities existed in the rural areas, but there was no difference in drinking days during pregnancy. 

### 3.3. Rates of Total FASD and by Specific Diagnoses

**Figure 3:**
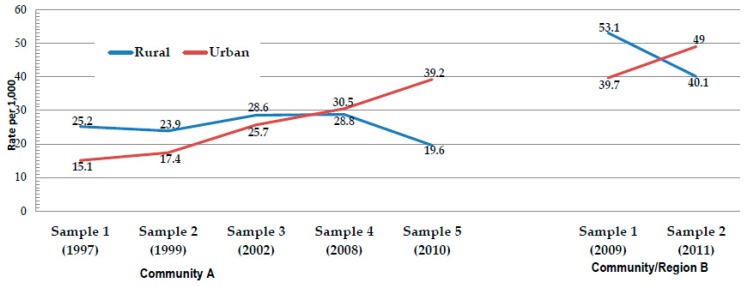
Rate of fetal alcohol syndrome (FAS) by rural and urban residence.

**Figure 4:**
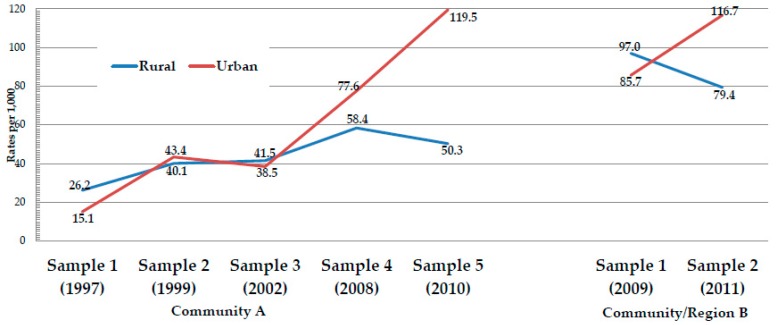
Rate of total FASD by rural and urban residence.

In Table 6, the rates (per 1000) of specific FASD diagnoses are presented by residence. In Samples 1 and 2 from Community A, the rate of FAS, the most severe form of FASD, was significantly higher in the rural areas. However, this was not the case in Sample 3 or Sample 4, for it was not significantly different between rural and urban areas. In Sample 5, the rural rate of FAS was significantly lower than the urban rate: the rural rate of FAS was 19.6 and the urban rate was 39.2. Since FAS, as the most severe FASD phenotype, was the primary diagnostic focus of each of these samples from the outset, the temporal pattern exhibited by the FAS rates is likely a true one and was not likely to have been influenced by improved diagnostic skills or enhanced diagnostic criteria among the clinical team. In Community A, there was a rise in the total FASD rate from Sample 1 to Sample 5, but the ARND diagnosis was not used in Samples 1–3, and it was only possible to assess this in a few cases retrospectively in Sample 2. See the illustrated trends for FAS rates in Figure 3, and for FASD in Figure 4. In Community B, there were fewer samples over a shorter period of time, but the rural/urban differences were also apparent in this community. In Community B, Sample 1, the rate of FAS was significantly higher in the rural areas (53.1) than in the urban areas (39.2), but in Sample 2, the pattern was reversed, with the urban rate being significantly higher (40.1 vs. 49.0). Partial FAS was significantly higher in the urban areas in every comparison in both communities. The rural/urban difference was statistically significant for ARND in three of the comparisons and ARND rates were consistently higher in the urban areas, with only an exception in Sample 1 of community B (see Table 6 and Figure 3 and Figure 4).

### 3.4. Association Analysis: The Direct Relationship between Heavy Drinking, FASD Diagnoses, and Dop Experience

In an attempt to place any direct contribution of Dop to maternal drinking and the prevalence of FASD into a quantitative perspective, partial correlations and sequential regression analysis were performed. Partial correlations of lifetime experience with Dop with drinks per drinking day (DDD) were performed while controlling for residency (rural/urban) and maternal age at index pregnancy. The Pearson product-moment correlation between ever receiving Dop in one’s lifetime with an average of 3 DDD or more was statistically significant, but extremely weak (*r* = 0.117, *p* < 0.001). The correlation between 5 DDD or more was also significant, but equally weak (*r* = 0.123, *p* < 0.001). Therefore, the associations between Dop and heavy drinking explained only 1.4%–1.5% of the variance in heavy drinking. Additionally, when the average DDD was added to the other two controlled variables above and the correlation of lifetime Dop with an FASD diagnosis was examined, the correlation was even weaker (*r* = 0.059, *p* = 0.010), explaining 0.35% of the variance in FASD diagnosis.

In Table 7, a sequential regression analysis is presented. Controlling for average DDD consumed, age, residence, gravidity during the index pregnancy, and maternal BMI, the average DDD is the largest contributor (β = 0.302, *p* < 0.001, odds ratio = 1.352, 95% CI = 1.296–1.410) to the diagnosis of FASD in the offspring. A low maternal BMI, rural residence during pregnancy, and higher gravidity at the index pregnancy all also contribute significantly. However, there is no significant, direct association between ever receiving Dop in one’s lifetime and an FASD diagnosis in the offspring (β = 0.304, *p* = 0.183, and an extremely small contribution to the cumulative odds ratio of 0.003 and 0.3% to the cumulative classification accuracy).

## 4. Discussion

### 4.1. The Dop System in Contemporary Western Cape Communities

These community-specific, time series data support the idea that the Dop system of partial payment for labor via the distribution of wine is now extremely rare in the WCP. Even lifetime rates of experience with Dop were found to be very low in the time-period studied. In the more recent time-periods sampled, an even lower percentage of women reported receiving Dop payments in their lifetime, currently, or during pregnancy. In the latest samples collected from the more remote and rural regions of the province, the lifetime prevalence of Dop payments in the two communities was 2.9%–17.5% in rural areas and 0.6%–8.5% in the urban areas. Dop is less commonly reported by those residing in Community A (a developing community closer to Cape Town), than among those living in more remote rural settings in Community B. By the time of the interview, seven years after the birth of the index children, virtually no Dop was being distributed (0.0%–0.6% in community A, and 0.0%–0.7% in community B). Moreover, these recent reports of Dop were provided by only a few mothers, all of whom had children diagnosed with an FASD. Therefore, Dop, as a large, common, and viable institution, is essentially dead in the WCP, and is currently reported as a source of alcohol by less than 1% of the mothers of children with an FASD. While none of the mothers of the randomly-selected children who were found to be functioning normally (controls) reported receiving any alcohol via the Dop system, as we have reported before, commercially-produced and purchased alcoholic beverages constitute virtually all of the alcohol consumed by women of a childbearing age in these communities today [14,15,16,22,23,28].

### 4.2. Has the Dop System Been Influential in Today’s Alcohol Use Patterns?

While the partial correlation analysis produced a very weak, but statistically significant, association (explaining <2% of the variance) in heavy maternal drinking, the more sophisticated sequential correlation analysis showed no statistically significant, direct relationship between Dop and an FASD diagnosis in the offspring. There is no real direct association between the current Dop distribution and heavy drinking or with the likelihood of an FASD diagnosis in the offspring. It should, furthermore, be noted that the proportion of women reporting drinking during pregnancy in this paper, 41.4%–74.4%, was exponentially higher than the 3.1% of women who had given birth in the past five years reporting that they had consumed alcohol while pregnant [7,33]. Additionally, in the United States, recent surveys indicate that 7.3% of surveyed women (10.4% of those 25–29 years of age) who knew they were pregnant reported the consumption of some alcohol in the previous 30 days [34]. Therefore, we must point out that other factors, such as those found in studies conducted in other parts of South Africa, and elsewhere, are also likely to play a role in driving heavy drinking and its subsequent impacts on health, including on the developing fetus of pregnant women who drink. These include industry marketing practices such as the packaging and sale of beer in above average container sizes, such as 1 liter bottles of beer; perceptions of availability of alcohol; and exposure to alcohol promotions and advertising through SMS and free offers when buying alcohol [7,33] in a recent South African national household survey [33]. It is unlikely that this enormous difference can be explained purely by the varying method of asking questions; that is, a detailed one-on-one interview only asking about drinking during pregnancy and an omnibus, household survey. 

The data presented in this paper also provide much insight into the historic pattern of partial payment for labor via the Dop system and how it has been influential in shaping drinking practices of women today. It has, therefore, been influential in shaping normative patterns of alcohol use and the prevalence and pattern of FASD diagnoses found in contemporary WCP communities today. Until very recently, the bulk of the cases of FAS and PFAS (the more severe forms of FASD) have been found in the rural areas where Dop practices were originally implemented, and heavier drinking is still reported by the rural respondents. Therefore, the relationship today is certainly an indirect one transmitted through normative drinking patterns of the Dop heritage to that of today. Other factors, such as the possibility of a cohort effect producing reduced drinking levels in the study areas independently of any effects of the Dop system, are unlikely and not supported by any data. Nevertheless, this cannot be completely excluded. While not much has been written on the effect of the Dop system and its legacy on male health outcomes, besides a paper by London et al. [2], the dependency on alcohol created by the legacy of the Dop system on males warrants more research. Furthermore, the effects of industry products and marketing [7,33] have no doubt created an environment in which male drinking behavior makes it harder for women who are pregnant to stop drinking. This is also a topic for future research.

First, the pattern of heavy drinking that is present today in the study area, particularly on weekends and in rural areas, appears to be an extension or progression of the old pattern practiced under Dop. Second, 93%–98% of the alcohol consumed by the female respondents in these samples is consumed, on average, on only two days during weekends. Third, DDD remains consistently higher in the rural areas of both regions. DDD averaged 3.9 to 5.5 in Community A, which was more urban, and 5.4 to 5.7 in the more remote and rural Community B. Even though an alcohol allotment was supplied each evening to male workers on some farms under the Dop system, binge drinking was also reported to be a common practice for recreational drinking among men and women under the Dop system on some farms. Such recreational drinking primarily occurred on weekends and holidays. Fourth, few mothers reported drug use until the most recent samples in both communities. There is no appreciable (statistically significant) pattern of increase or decline in drug use reported in Community A over the 15 years studied, and the two samples in Community B were too close together to depict a definitive trend. Given that alcohol is still the drug of choice, the contemporary pattern of drug choice is similar to that of Dop. Fifth, drinking patterns remain more severe in the rural areas, which may be a Dop residual, and the most lifetime experience with Dop is reported by mothers of children with FASD. Sixth, high rates of total FASD have shifted from rural to urban areas in both communities. Although FAS rates have gone down overall, a relative upturn has now been registered in the urban rates in Community A over time. In Community B, the same trend may be emerging. Sixth, lifetime experience with Dop was significantly higher among mothers of children with FASD. Therefore, Dop practices appear to have been influential in directly encouraging heavy drinking that led to FASD in 10%–16% of the mothers in these study communities, especially in the earlier years in Community A, and in 2009 and 2011 in the more isolated Community B. Seventh, and lastly, rates of FAS have declined in the rural areas of Community A and appear to be declining in rural areas of Community B. FASD rates have increased overall in both communities over time, but part of this overall increase is likely due to two factors: increased access to relatively inexpensive, commercially-produced alcohol and the better diagnosis of less severe diagnoses with the FASD continuum (partial FAS and ARND). In the most recent samples of both communities, total FASD rates are increasing and higher in the urban areas. Therefore, each of these seven contemporary patterns of alcohol consumption seems to mirror patterns developed in the era of Dop. 

At least two phenomena may be at work to create the change in the rural and urban distribution of FASD cases over time. Community efforts at FASD prevention were initiated in 1998 in Community A. A National Institute on Alcohol Abuse, and Alcoholism (NIAAA)-funded comprehensive prevention program was also initiated in Community A in 2008 by the NIAAA research and prevention team. In the latter program, much emphasis was placed on awareness training, case management of high-risk women, and targeted education on many of the farms of the rural areas. Prevention efforts may have reduced the heaviest drinking among a significant number of rural women, which in turn may have lowered the severity of FASD characteristics and traits, thereby reducing the rate of FAS, and possibly total FASD among rural dwellers [14,23,35]. Furthermore, a pattern of residential relocation from the farms to the towns began in the mid 2000’s, and the most problematic drinkers may have moved or been moved to the urban areas where the supply of alcohol was greater.

One major difference in the drinking style practiced in the Western Cape is a change of beverage. In the days of Dop, the alcoholic beverage most commonly consumed was wine, but in this study period, the beverage of preference was beer. The most frequently purchased beverage was higher alcohol-content beer (approximately 5.5% in the most popular brand) [14,22]. Furthermore, there is a recent trend towards groups purchasing and consuming multiple, high-alcohol-content beverages (fortified wine, rums, vodka, etc.) for consumption on drinking evenings. This latter trend is concerning.

## 5. Limitations and Strengths of the Study

First, the data used in this targeted study were initially collected to determine the prevalence and characteristics of FAS and other FASD in these communities. Therefore, not all Dop questions were asked in the first sample of Community A. However, in all subsequent samples, the full battery of three Dop questions was asked. Second, the study was limited to covering only females of a childbearing age, and the Dop questions were not asked to men, specifically the fathers of the study children. It could be that male workers in the agricultural system were more likely to receive Dop distributions. However, from the information provided by our field workers, we are not aware that this is the case. Third, we know that over the last three or four decades, the majority of farmers and their families are knowledgeable and concerned businessmen and women. Many have increasingly incorporated improvements in permanent residential and working conditions, and the lives of workers have substantially improved on the farms [5]. Where there is less concern for the welfare of the workers, and less tolerance of heavy drinkers, many workers have moved or been moved into the towns where alcohol supplies are greater. We were not able to address this migration trend with our data. 

## 6. Conclusions

The systematic process of Dop is virtually gone or dead, but the legacy of alcohol as the recreational drug of choice remains. Furthermore, the pattern of heavy drinking among men and women is, in many ways, similar to the weekend pattern during the days when Dop was a relatively common institution in WCP. Dop seems to have created a common, culturally-supported pattern of the heavy consumption of commercially purchased alcoholic drinks practiced by most men and many women that has been translated into contemporary practices and, therefore, led to continuing high rates of FASD in these communities. 

## Figures and Tables

**Table 1:** Alcohol, tobacco, and other drug use patterns in seven samples in two separate regions.

	Community A												Community/Region B					
	Sample 1 [1997] (*n* = 86)		Sample 2 [1999] (*n* = 211)		Sample 3 [2002] (*n* = 222)		Sample 4 [2008] (*n* = 303)		Sample 5 [2010] (*n* = 466)		Test-Score	*p*	Sample 1 [2009] (*n* = 670)		Sample 2 [2011] (*n* = 450)		Test-Score	*p*
**Current Alcohol Consumption**																		
Drank in past 12 months (% Yes)	58.1		53.0		41.6		48.2		56.0		χ^2^ = 15.532	0.004	54.3		66.6		χ^2^ = 14.902	<0.001
Drank in past 30 days (% Yes)	58.1		50.9		28.4		38.5		41.6		χ^2^ = 32.505	<0.001	42.6		50.2		χ^2^ = 6.348	0.012
Drank in the past week (% Yes)	58.1		34.5		22.3		32.5		30.0		χ^2^ = 38.301	<0.0001	34.1		38.7		χ^2^ = 2.466	0.116
Total # of drinks per week ^1^–Mean (SD)	11.7	(10.5)	12.9	(13.7)	8.1	(8.8)	8.1	(8.8)	9.6	(9.6)	F = 3.110	0.015	10.2	(9.4)	10.1	(7.5)	t = 0.057	0.955
# of drinking days per week ^1^	1.9	(0.9)	1.9	(1.5)	1.9	(1.0)	1.8	(1.0)	1.8	(1.2)	F = 0.303	0.876	1.8	(1.1)	1.8	(1.0)	t = 0.534	0.811
Drinks per drinking Day^1^ (DDD)	5.5	(3.6)	5.4	(3.9)	3.9	(2.8)	4.2	(3.0)	4.3	(2.8)	F = 3.106	0.016	5.7	(5.2)	5.4	(3.2)	t = 0.798	0.425
# drinks consumed on (Friday–Sunday)^1^	11.3	(10.4)	11.9	(11.5)	8.1	(8.7)	8.5	(9.1)	9.1	(8.7)	F = 0.801	0.525	11.3	(18.4)	10.1	(7.3)	t = 1.643	0.101
**Beverage of choice (% Yes) ^1,2^**																		
Beer	54.0		78.4		81.6		71.6		79.1		χ^2^ = 15.009	0.005	81.5		82.8		χ^2^ = 0.106	0.744
Fortified Wine	0.0		0.0		4.1		2.1		0.0		χ^2^ = 8.955	0.062	2.2		4.6		χ^2^ = 1.801	0.180
Spirits	0.0		4.1		6.1		16.8		8.8		χ^2^ = 15.537	0.004	9.3		15.5		χ^2^ = 3.670	0.055
Wine	8.0		36.5		53.1		29.5		32.4		χ^2^ = 24.211	<0.001	46.3		48.9		χ^2^ = 0.266	0.606
Combination	38.0		1.4		4.1		2.1		1.4		χ^2^ = 98.292	<0.001	3.5		3.4		χ^2^ = 0.002	0.967
**Past alcohol consumption**																		
Drank during pregnancy (% Yes)	74.4		41.4		48.4		49.9		58.1		χ^2^ = 35.838	<0.001	57.7		67.5		χ^2^ = 11.376	0.001
**Tobacco and Other Drug Use**																		
Used tobacco in lifetime	75.6		46.9		63.0		70.3		63.4		χ^2^ = 49.050	<0.001	70.0		67.6		χ^2^ = 0.782	0.377
Use tobacco in past 30 days ^3^	83.1		83.2		77.0		91.1		79.7		χ^2^ = 14.094	0.029	81.9		77.3		χ^2^ = 2.347	0.126
# of cigarettes per day–Mean (SD)	9.8	(7.7)	5.1	(5.2)	4.7	(3.7)	5.8	(5.0)	6.4	(4.9)	F = 8.536	<0.001	4.9	(4.2)	8.8	(12.5)	t = -4.279	<0.001
Used drugs–past year (% Yes)	--		0.5		0.4		0.7		1.9		χ^2^ = 4.548	0.208	0.6		1.5		χ^2^ = 2.623	0.105
Used drugs–pregnancy (% Yes)	--		0.0		0.9		0.7		1.5		χ^2^ = 4.787	0.310	1.0		2.8		χ^2^ = 5.33	0.201

^1^ Includes only those who consumed alcohol in the previous 7 days. ^2^ Some respondents specified more than one beverage of choice. ^3^ Includes only those who have ever used tobacco in their lifetime. No post-hoc Dunnett C comparisons for Community A were significant at the 0.05 level. Bonferroni-adjusted significance values: whole table < 0.003; current alcohol consumption < 0.007; other sections < 0.005.

**Table 2:** Percent receiving Dop in their lifetime.

	Community A							Community B			
	Sample 1 [1997] (*n* = 86)	Sample 2 [1999] (*n* = 211)	Sample 3 [2002] (*n* = 222)	Sample 4 [2008] (*n* = 303)	Sample 5 [2010] (*n* = 466)	χ^2^	*p*	Sample 1 [2009] (*n* = 670)	Sample 2 [2011] (*n* = 450)	χ^2^	*p*
**Received Dop in Lifetime (%Yes)**	--	5.7	5.9	2.3	1.1	17.435	0.001	11.0	11.8	0.144	0.704
**Received Dop in Pregnancy (%Yes)**	--	1.5	0.5	1.0	0.4	2.585	0.460	0.9	0.2	1.950	0.163
**Received Dop at time of interview**	0.0	0.5	0.0	0.3	0.2	0.113	0.774	0.3	0.2	0.054	0.816

--Question not asked in Sample 1; Not all Dop questions were asked in the first sample of Community A.

**Table 3:** Percent of mothers receiving Dop by diagnosis of children and different time periods: Lifetime, during pregnancy, and at time of interview.

	Community A										Community/Region B			
	Sample 1 [1997]		Sample 2 [1999]		Sample 3 [2002]		Sample 4 [2008]		Sample 5 [2010]		Sample 1 [2009]		Sample 2 [2011]	
	FASD	Controls	FASD	Controls	FASD	Controls	FASD	Controls	FASD	Controls	FASD	Controls	FASD	Controls
**Received Dop in Lifetime (% Yes)**	--	--	11.5	2.8 *	9.5	7.3	5.9	0.0 ***	1.6	0.7	16.4	6.4 ***	15.8	6.5 **
**Received Dop in Pregnancy (% Yes)**	--	--	3.2	0.8	1.6	0.0	2.5	0.0	1.1	0.0	2.0	0.0**	0.4	0.0
**Received Dop at time of interview**	0.0	0.0	1.6	0.0	0.0	0.0	0.8	0.0	0.6	0.0	0.7	0.0	0.4	0.0

Chi-square comparisons: * *p* < 0.05; ** *p* < 0.01; *** *p* < 0.001. --Question not asked in Sample 1.

**Table 4:** Percent of mothers receiving Dop by current maternal age and different time periods: Lifetime, during pregnancy, and at time of interview.

	Current Maternal Age 20–29	Current Maternal Age 30–39	Current Maternal Age 40+	*p*
**Received Dop in Lifetime (% Yes)**	2.8	6.8	13.7	<0.001
**Received Dop in Pregnancy (% Yes)**	0.5	0.6	1.4	0.134
**Received Dop at time of interview (% Yes)**	0.5	0.1	0.4	0.300
**Rural residence (% Yes)**	36.7	34.7	38.5	0.276

**Table 5:** Rural and urban drinking levels by lifetime, year, months, weeks, days, weekends, and during pregnancy.

	Rural		Urban		*p*
**Drank in Lifetime (% Yes)**	91.8		79.5		<0.001
**Drank in past 12 months (% Yes)**	58.2		52.7		
**Drank in past 30 days (% Yes)**	49.1		39.7		<0.001
**Drank in the past week (% Yes)**	42.6		28.9		<0.001
**Total # of drinks per week ^1^–Mean (SD)**	12.3	(15.9)	8.4	(7.9)	<0.001
**# of drinking days per week ^1^–Mean (SD)**	1.9	(1.0)	1.8	(1.2)	0.108
**Drinks per drinking day^1^ (DDD)–Mean (SD)**	5.9	(4.7)	4.4	(2.9)	<0.001
**# of drinks consumed on (Friday** **–Sunday) ^1^**	11.6	(12.4)	7.6	(7.0)	<0.001
**Drank during pregnancy (% Yes)**	70.6		49.2		<0.001

^1^ Calculated only for those who reported drinking in the past year.

**Table 6:** Rates (per 1000 children) of specific fetal alcohol spectrum disorders (FASD) diagnosis and total FASD by rural and urban residence: Western Cape Province communities: 1997–2011.

	Community A															Community/Region B					
	Sample 1			Sample 2			Sample 3			Sample 4			Sample 5			Sample 1			Sample 2		
	Rural	Urban	*p* ^^^	Rural	Urban	*p* ^^^	Rural	Urban	*p* ^^^	Rural	Urban	*p* ^^^	Rural	Urban	*P* ^^^	Rural	Urban	*p* ^^^	Rural	Urban	*p* ^^^
**FAS**	25.2	15.1	<0.001	23.9	17.4	<.0001	28.6	25.7	0.142	28.8	30.5	0.737	19.6	39.2	<0.001	53.1	39.7	<0.001	40.1	49.0	<0.001
PFAS *	1.0	3.0	0.002	13.0	18.4	0.001	4.9	12.8	<0.001	14.8	30.5	<0.001	19.6	58.0	<0.001	27.3	31.1	<0.001	25.6	43.5	<0.001
ARND **	0.0	0.0	--	3.3	7.6	<0.001	0.0	0.0	--	14.8	16.6	0.236	11.1	22.2	<0.001	16.6	15.0	<0.001	13.8	24.2	<0.001
**Total FASD**	26.2	18.1	<.001	40.1	43.4	0.151	41.5	38.5	0.168	58.4	77.6	<.001	50.3	119.5	<0.001	97.0	85.7	<0.001	79.4	116.7	<0.001

* PFAS cases in Samples 1 and 2 were made retrospectively in 2003 as the diagnostic criteria were clarified. The focus at the time of examination for Samples 1 and 2 was exclusively on FAS. ** No ARND cases were diagnosed in Samples 1 and 3 because of the singular focus on FAS. The ARND cases diagnosed in Sample 2 were diagnosed retrospectively, and not at the study, as the diagnostics interview for ARND was clarified by 2008. ^z-test of proportions.

**Table 7:** Sequential regression to estimate the association between lifetime experiences with the Dop distribution system and a diagnosis of FASD in the offspring.

	*β*	S.E.	Sig.	Odds Ratio	95% C.I. for Odds Ratio	
					Lower	Upper
***Covariates***						
Estimated average # of drinks consumed per day in pregnancy	0.302	0.022	0.000	1.352	1.296	1.410
Age at time of pregnant with COI (in years)	0.007	0.009	0.443	1.007	0.989	1.026
Urban or rural residence during pregnancy	−0.280	0.114	0.014	0.756	0.605	0.945
Maternal BMI	−0.048	0.008	0.000	0.953	0.938	0.969
Gravidity	0.124	0.043	0.004	1.132	1.040	1.232
***Predictor***						
Ever receive Dop–in lifetime	0.304	0.228	0.183	1.355	0.866	2.121
Constant	−0.119	0.313	0.702	0.887		

Drinks per drinking day (DDD) predicted diagnosis (χ^2^ = 462.325, *p* < 0.001, Nagelkerke R^2^ = 0.293). Addition of demographic covariates predicted diagnosis (χ^2^ = 64.507, *p* < 0.001, Nagelkerke R^2^ = 0.328). Addition of Dop (ever in lifetime) (χ^2^ = 1.785, *p* = 0.183, Nagelkerke R^2^ = 0.329).
